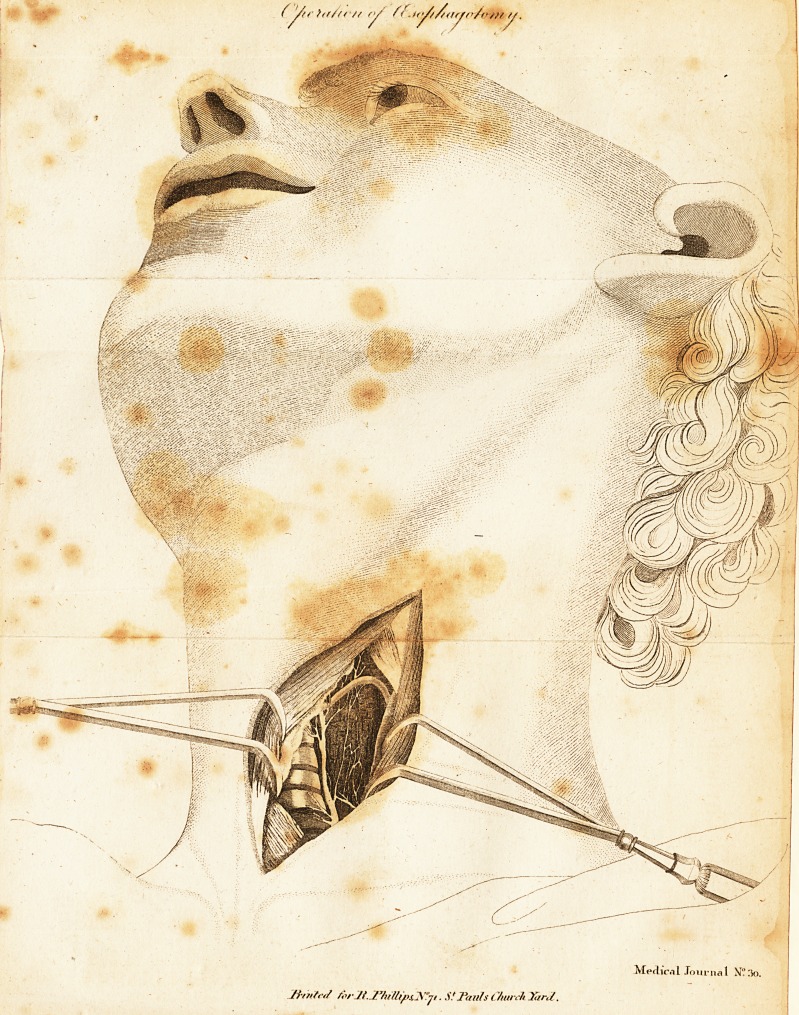# A Description and Engraving of the Operation of Oesophagotomy

**Published:** 1801-08

**Authors:** William Blair

**Affiliations:** Member of the Royal College of Surgeons in London, Surgeon of the Lock Hospital and Asylum, of the Finsbury Dispensary, and of the Bloomsbury Dispensary, &c.


### Medical and Physical Journal.

VOL. VI.] AUGUST, 1801. [NO. XXX.

### *A Description and Engraving of the Operation of Oesophagotomy, by* WILLIAM BLAIR, A. M. F. M. S.

Member of the Royal College of Surgeons in London, Surgeon of the Lock Hospital and Asylum, of the Finsbury Dispensary, and of the Bloomsbury Dispensary, &c.

ALTHOUGH the operation of making a longitudinal incision into the œsophagus, for the purpose of extracting some foreign substance threatening the death of the animal, has been performed with success on various brute creatures, (especially the cow, the horse, and the dog,) it has always been regarded by surgeons as a very important and hazardous undertaking on the human subject. The first hint I find relating to this operation, is contained in the second volume of *Pathologie de Chirurgie, par Jean Baptiste Verduc*, who, in treating of the mode of extracting foreign bodies from the œsophagus, says, if you cannot succeed, this operation may be had recourse to. "Je crois qu'on pourra fort bien hazarder l'operation, en faisant une incision à l'œsophage pour avoir ce corps étranger. On fera la même chose qu'à la bronchotomie; il faudra d'abord séparer les muscles bronchiques, pour aller d'une main adroite chercher l'œsophage, et faire une incision longitudinale à l'endroit du corps étranger. Je vous avouë que cette operation est difficile; mais il vaut mieux l'entreprendre, que d'avoir le déplaisir de voir mourir le malade."

The above description is too vague and concise to guide the inexperienced surgeon. It does not appear that M. Verduc himself ever performed the operation; but an eminent practitioner of Rome has left us more explicit directions, in the *Memoires de l'Académie Royale de Chirurgie, Tome iii. p.* 351, 4*to edit. Essai sur l'Oesophagotomie, par* M. GUATTANI. In another work of VERDUC, indeed, we are told that this operation has been performed by several practitioners with the most happy event. Vide *Abreg. compl. de la Chirurg. de* GUY de CHAUL. *chap. sing. artic. de l'Exerese; et Mem. de l'Acad.*

*de Chir. Tome i. p.* 590. Various examples* are alſo on record of patients having recovered, after accidental wounds of the œſophagus had been inflicted; ſo that we are ſufficiently authoriſed to have recourſe to this method of relief, in caſes of imminent danger.

M. Guattani obſerves that it is neceſſary, in order to perform this operation properly, to know what is the relative ſituation of the œſophagus. It was noticed, he ſays, by Euſtachius, afterwards by Veſalius, by Winſlow, by Haller, &c. that the œſophagus lies a little on the left ſide of the trachea; this was always found to be its poſition in the many bodies diſſected by Guattani. So important a fact, now well eſtabliſhed, ought to guide us in performing the operation. He therefore directed it to be done in the following manner:

The patient being placed upon a chair, let an aſſiſtant bend his head ſtraight backwards, and ſecure it in ſuch a way as to render every lateral motion of it impoſſible. The operator then places himſelf immediately before the patient; and having, with his left hand, drawn the ſkin tight into a tranſverſe fold, on the right ſide of the neck, which is alſo done on the left ſide by an aſſiſtant, he divides the integuments of the neck with a ſtraight ſcalpel, longitudinally, from the upper part of the trachea down to the ſternum. He now ſeparates the cellular, adipoſe, and membranous parts, in ſhort, whatever appears between the ſterno-thyro-hyoid muſcles and above the trachea, with the ſame ſcalpel; which he then puſhes, on the left ſide, (as the œſophagus generally projects more on this ſide than on the other) deeper in, between the above-mentioned muſcles and the trachea; whilſt, at the ſame time, in order to obtain room, the lips of the wound are drawn aſunder by means of two double-pronged hooks. The deeper-ſeated cellular ſubſtance, ſurrounding the trachea, he ſeparates with his finger, or, if neceſſary, with the knife, till the œſophagus is brought diſtinctly into view, which he then opens longitudinally, beginning from below; and where the circumſtances require it, he enlarges the wound at the upper part with a crooked pair of ſciſſars.

After the operation has been conducted thus far, Guattani directs us to uſe curved forceps, if they be wanting, to extract the foreign ſubſtance; or, if that be not practicable, it may be puſhed down to the ſtomach. Any hæmorrhage that occurs may be ſtopped by preſſure with the finger, or by the ligature; and

* By Parey, De la Faye, Munnicks, Habicot, Pigray, Garengeot, Schenkius, Bohnius, Poncenard, Bell, &c.

and the recurrent nerve may be avoided, with care. The wound will heal without much difficulty, and is to be dreffed in the moft fimple manner poffible. During the cure we muft keep the patient on the loweft diet, or rather, fupport him with nourifhing clyfters.

This account of M. Guattani is accompanied with a detail of fome experiments, tending to fanction his practice; and the Academy of Surgery, to whom he prefented the Memoir, have feemed to adopt his ideas, by joining to his Memoir an account of two cafes of Oefophagotomy, practifed fuccefsfully, after his method, on living men. Thefe operations were performed by M. M. GOURSAUD and ROLAND, *Hiftoire de l'Acad. Roy. de Chir. Tome* III. *p.* 13. vii. The fame method is advifed to be purfued by SABATIER, *de la Med. Operat. Tome* III. *p.* 471; alfo by M. M. CHOPART and DESAULT, *Traité des Mal. Chir. et des Oper. Tome* I. *p.* 273: likewife by BERTRANDI, who fays he has feveral times done it fuccefsfully on dogs, *Trattato delle Operazioni di Chirurgia, Cap.* xx. NANNONI, a late Italian furgeon, and Mr. B. BELL, in their refpective fyftems, have alfo commended the method of Guattani.

The only objections I have met with againft this operation, have been recently brought forward by a German furgeon, who propofes feveral deviations and improvements: *Ueber das Ausziehen fremder Körper, aus dem Speisekanle und der Luftröhre, von* J. D. ECKHOLDT, M. D. & Chir. &c. Kiel, 1799. This author has written the moft elaborate work now extant, accompanied with fplendid engravings, on all the means ufed for removing extraneous bodies from the œfophagus and trachea. I fubjoin what he fays on the prefent fubject, and have annexed a copy of his engraving of the operation.

The opening of the œfophagus, he obferves, is indicated, in the firft place, when the foreign fubftance, which, either on account of a fpafmodic affection, or the tumefied ftate of the parts above it, or owing to the incompetency of our inftruments, cannot be extracted upwards, is either difficult of folution, or altogether infoluble. Secondly, when it is of a pointed or angular form, which affords caufe to apprehend a violent inflammation, not only in the œfophagus, but alfo in all the reft of the alimentary canal, in cafe it fhould reach the ftomach. Thirdly, when its fize is fo large that we have no reafon to hope that it will be able to pafs through the alimentary canal without danger; in which cafe it commonly happens that, even during its ftay in the œfophagus, it produces fuch a preffure upon the trachea, as fometimes to render neceffary both operations, namely, the fection of the œfophagus and that of the trachea.

 As

As a counter-indication to this operation, among others, the cafe in which the foreign fubftance is fituated very low down in the alimentary canal, is generally mentioned. However, the force of this counter-indication is done away by the invention of the author's extracting inftrument, which may be introduced equally well, either through the wound or the mouth.

Great debility of the patient has alfo been confidered a counter-indication, though with what right he cannot comprehend, as it is every phyfician's duty to do what he can, as long as there remains the fainteft hope that the patient's life may be preferved. At any rate, the confequence of the oppofite practice is inevitable death.

Another, and Mr. Eckholdt confeffes a very important, counter-indication is generally mentioned; namely, the too great inflammation and tumefaction that has taken place in the œfophagus and neighbouring parts. This, however, he cannot admit unconditionally; nay, he maintains, on the contrary, that fuch a ftate of the parts ought by no means to deter the furgeon from performing the operation, but rather induce him to undertake it the fooner the better. For the very circumftance, which in fuch a cafe is moft apprehended, namely, the injury that may be done to confiderable blood-veffels and nerves, is here not fo eafily poffible; for thefe parts, which of themfelves undergo a proportionally lefs alteration than the reft, are more difplaced from their natural fituation, and removed farther from each other, by means of the inflammatory fwelling, on which account they are far lefs expofed to the danger of being cut during the operation. Moreover, under fuch circumftances, the cutting of the other inflamed parts is not only not detrimental, but rather beneficial, on account of the difcharge of blood which it occafions from the fmaller veffels.

This may be fufficient concerning the counter-indications that have commonly been adduced againft this operation. The author obferves, that the fection of the œfophagus is an operation hitherto almoft univerfally dreaded by furgeons. In order to afcertain whether this dread be well or ill founded, we muft trace it to its caufe, which, from what has already been faid, appears to depend partly upon the danger of injuring confiderable blood-veffels and the recurrent nerves of the eighth pair, and partly upon the difficulty with which the cure of the wound is attended. The dividing of the firft may produce fatal confequences; and that of the laft, if it be done on both fides, the entire lofs of voice; or if only on one, a great feeblenefs of it, as we may frequently fee in the examples of perfons who have attempted to cut their throats.

It

It certainly cannot be denied that there is no ſpot on the human body in which there are ſo many ſingle parts, the leſion of which may be productive of very ſerious inconveniencies, contained within a ſmaller ſpace, than the neck; it is therefore very neceſſary that great caution ſhould be employed, not only in chooſing the place where ſuch an inciſion is to be made, but alſo in performing the operation itſelf. However, our ſureſt guide, experience, ſupported by the anatomical knowledge of this part, has proved that a cautious and ſufficiently experienced operator may undertake the ſection of the œſophagus without apprehenſion. Even the variations of the forms of different parts, which but too often occur, need not deter the ſurgeon, and ſtill leſs ſhould they embarraſs him in performing the operation; for he can and muſt know what he ought to do in ſuch caſes.

After deſcribing Guattani's operation, the author ſays, this method of operating is attended with ſeveral inconveniencies, which the ſurgeon can never entirely avoid, unleſs he chooſes another and more convenient place for performing the operation. The firſt great difficulty which here preſents itſelf, is, that it is impoſſible for us, by drawing aſunder the trachea and the muſcles which cover it, to obtain ſo much room as we require in order to uſe the knife properly, and conveniently to extract the foreign ſubſtance. The ſhortneſs of the muſcles in queſtion, their ſituation, the more rigid and leſs yielding ſtructure of the trachea, do not permit thoſe parts to be drawn aſunder to the degree that is requiſite for the accompliſhment of our intended purpoſe. Moreover, in operating at this part, we are naturally expoſed to the danger, (which is increaſed under the above mentioned circumſtances) of injuring the inferior thyroid artery and the recurrent nerve; eſpecially the latter, which, in moſt ſubjects, aſcends towards the larynx exactly at the place where the inciſion into the œſophagus is to be made. Now, how is it poſſible to avoid this nerve in ſo confined a ſpace? Another no leſs important obſtacle is the thyroid gland, whoſe ſize is not always ſuch that it can be entirely ſpared. The leſion of it, indeed, is of itſelf attended with no material danger; but the violent hæmorrhage which it produces, even though it ſhould not influence the ſucceſs of the operation, however, retards the ſurgeon, to the great diſadvantage of his patient. Finally, the moſt weighty objection that can be adduced againſt the place choſen for performing this operation is, that the re-union of the wound, whoſe ſuperior or external part has more tendency to re-unite than that which is deeper ſituated, can never be accompliſhed in a perfectly uniform manner; conſequently, in the firſt ſtage of the cure, whilſt whilſt the wound of the œſophagus has not yet cloſed, and the ſpace above it is not yet filled up, can ſcarcely be kept open without the application of ſome degree of force.

Induced by theſe conſiderations, which will certainly be allowed to have their weight, Mr. Eckholdt fixed upon another place for performing the operation in queſtion, which he now propoſes and recommends without fear of contradiction, as the moſt eligible.

The manner in which he performs the ſection of the œſophagus is as follows:

The patient being placed upon a bed, in as unconſtrained a poſture as poſſible, his neck reſting upon a ſmall bolſter ſtuffed with hops or chopped ſtraw, and his head bent backwards and inclined to the right ſide, in which poſture he is ſecured by an aſſiſtant, another aſſiſtant is directed to collect the ſkin, on the left ſide of the neck, over the muſcle that runs from the maſtoid proceſs to the clavicle and ſternum, at the place where the ſeparation of its inferior crura commences, into a ſomewhat oblique tranſverſe fold; in d ing which the operator aſſiſts him, at a ſmall diſtance, with the fingers of his left hand. He then, with a convex knife, cuts through this ſtretched fold of the ſkin, together with the broad muſcle of the neck ſituated immediately under it, and in the ſame manner enlarges the wound downwards, as far as the anterior articulation of the clavicle with the ſternum. It is eſſentially requiſite that this inciſion ſhould be made exactly in the middle of the above mentioned muſcle, and that it ſhould run in the ſame oblique direction from above to below, as is peculiar to this muſcular part. When this firſt inciſion has been made, there preſents itſelf to our view that triangular ſpace, which is formed in this part by the diviſion and courſe of the two inferior crura of the above mentioned muſcle, and the muſcle which runs above, at the beginning of this diviſion, obliquely acroſs from the os hyoides to the ſcapula. It is this which affords the ſurgeon acceſs to the œſophagus, and renders the operation eaſy and ſecure. For without being under the neceſſity of uſing any cutting inſtrument, he may now eaſily arrive at the œſophagus, by deſtroying the very looſe cellular ſubſtance which connects the two crura of the muſcle and fills up the ſpace above, with the flat handle of his knife, but the deeper ſituated cellular ſubſtance with his fore finger, ſo as at the ſame time to undermine the thyroid gland at the ſide. This being done, he examines whether the ſpace be wide enough to afford a ſufficiently diſtinct view of the œſophagus and the recurrent nerve of the eighth pair, which runs along its anterior ſide, next to the trachea. This is not always the caſe, as in ſome ſubjects the diviſion division of the muscle does not commence till very low down, whereby this space is rendered something narrower: Under such circumstances we must divide this muscle a little farther up; taking care, however, not to cut the muscle which goes from the os hyoides to the scapula, and which is situated in a cross direction under the former muscle. In order to avoid this accident, we should cut gradually from without to within, not from below to above. When I have either found the space sufficiently wide, or rendered it so by the operation just described, I direct the two edges of the wound to be drawn asunder, as far as it can be done without violence, by means of two crooked double-pronged hooks, whereby are exposed to view (as may be seen in the PLATE) at the back part, the great cervical artery, which does not project much; at the fore part, a portion of the thyroid gland and the trachea situated under it; at the upper part, the middle tendinous portion of the muscle situated between the os hyoides and the humerus; at the lower part, a portion of the muscle that goes from the thyroid cartilage to the sternum; and in the middle, the œsophagus, together with the inferior thyroid artery, which runs across it in several circumvolutions, and the recurrent nerve. The two sides of the wound being now perfectly secured, and drawn asunder by the two hooks, a longitudinal incision must be made into the œsophagus with the requisite caution, taking care not to injure the above-mentioned nerve or the thyroid artery; and this incision the operator enlarges by means of a crooked pair of scissars, as far as he has room; for the sides of a small wound may easily suffer by contusion in extracting a foreign body.

Should it be objected, that in this method of performing the section of the œsophagus, a deviation from the common structure of the parts, which could not be discovered from any external appearance, might entirely alter the case, and render this method of operation equally dangerous with that hitherto in use; M. Eckholdt answers, that such a circumstance, provided only that the deviations be not too considerable, and confine themselves merely to the situation of those parts which we are obliged to avoid, can be of no very material consequence. For as, after the first incision through the external integuments, there is no necessity for using the knife till we come to open the œsophagus, every thing that lies in our way may easily be spared; and, should even in any case the recurrent nerve be situated more upon the middle part of the œsophagus, it might, in such a free space, be easily discovered, and drawn aside, by means of a blunt hook, from the place where the incision is to be made. The same applies also to the large vessels, when they are situated too far forward; and to the inferior thyroid artery, artery, when feated deeper than ufual, efpecially at the place where it forms its laft inflexion. The latter, alfo, fhould it not be practicable to draw it afide, might, in cafe of neceffity, be tied without difficulty, and divided without danger.

He does not think it neceffary to fay any thing more concerning the extraction of the foreign fubftance, after the œfophagus has been opened, than that, if it be fituated near to the wound, the forceps he has delineated fhould be employed; but if it be fituated lower down, we fhould ufe the other inftruments defcribed as adapted for the purpofe; and that we fhould be as cautious as poffible to avoid any contufion of the lips of the wound in the œfophagus.

When the foreign fubftance has been extracted, our firft bufinefs muft be to endeavour to promote a proper and accurate reunion of the wounded parts. This the furgeon fhould confider as a matter of equal importance with the operation itfelf. Should any one think flightly of it, let him only recollect what has already been faid, and confider what a material influence a bad cicatrization of the œfophagus muft have upon its functions. Guattani, as well as others, have fhewn by experiment, that a wound of the œfophagus may eafily be cured, and without its edges forming any adhefions with the neighbouring parts, provided it be treated according to the rules which the art prefcribes. Let it therefore be our particular endeavour to bring its edges, at every point, into accurate and uniform mutual contact, and to retain them in this fituation during the firft days after the performance of the operation. This may fometimes be accomplifhed by means of graduated compreffes applied to both fides of the external wound, and a bandage, which keeps the head inclined towards the oppofite fide. But in cafes where the lips of the wound of the œfophagus have, during the extraction of the foreign fubftance, been much ftretched, bruifed, or even lacerated, we are obliged to have recourfe to the quill-future, which is preferable to every other, becaufe it can not only be applied and removed with greater facility than any other, but alfo poffeffes the advantage of leaving the wounded part of the œfophagus ftill fufficiently in our command. This may eafily be performed by means of a fmall needle cafe; but we fhould be careful not to twift the ends of the threads that hang out too tight, nor to fuffer the neck to be ftretched whilft we are applying the dreffings to the external wound.

In the author's opinion, the cure of the external wound fhould be left to the efforts of Nature till the fourth day, when the threads may be drawn out with fecurity; it fhould, therefore, only be covered loofely with lint, and a light comprefs, which which may be kept moist with some proper liniment, and secured by means of a circular bandage. At the same time we should endeavour, as has already been observed, to keep the head constantly inclined towards the opposite side, by means of another appropriate bandage. That invented by the late Dr. Koehler, of Jena, and delineated in plate VII. fig. 2, let. A, of his Treatise on Bandages, appears to be the best adapted for this purpose.

As to the treatment of the patient after the operation, besides the general remedies usually employed in such cases, and what will naturally suggest itself to the mind of the judiciouse practitioner, it is absolutely necessary, that for several days the patient should not be permitted to swallow any thing but liquids, in order that the fresh united edges of the wound may not be again torn asunder by any violent motion of the œsophagus For my part, says the author, I do not allow my patients to swallow even liquids, as long as I am not convinced that the canal has perfectly cicatrized; but during the first period of the cure, employ merely nourishing clysters, or, should it be necessary, nutritive baths. He adds, I am firmly convinced, as I have already asserted, that in the deglutition of fluids, the œsophagus exerts itself in a no less, if not more, violent manner, than in swallowing food of a pulpy or solid consistence. This I might demonstrate from repeated experiments which I have made, were I permitted to do so by the bounds I have allotted to this work.

## Figures and Tables

**Figure f1:**